# Topic Modeling Reveals Distinct Interests within an Online Conspiracy Forum

**DOI:** 10.3389/fpsyg.2018.00189

**Published:** 2018-02-21

**Authors:** Colin Klein, Peter Clutton, Vince Polito

**Affiliations:** ^1^School of Philosophy, The Australian National University, Canberra, ACT, Australia; ^2^ARC Centre of Excellence in Cognition and its Disorders, Macquarie University, Sydney, NSW, Australia; ^3^Department of Cognitive Science, Macquarie University, Sydney, NSW, Australia

**Keywords:** conspiracies, conspiracy theorists, topic models, social media, reddit

## Abstract

Conspiracy theories play a troubling role in political discourse. Online forums provide a valuable window into everyday conspiracy theorizing, and can give a clue to the motivations and interests of those who post in such forums. Yet this online activity can be difficult to quantify and study. We describe a unique approach to studying online conspiracy theorists which used non-negative matrix factorization to create a topic model of authors' contributions to the main conspiracy forum on Reddit.com. This subreddit provides a large corpus of comments which spans many years and numerous authors. We show that within the forum, there are multiple sub-populations distinguishable by their loadings on different topics in the model. Further, we argue, these differences are interpretable as differences in background beliefs and motivations. The diversity of the distinct subgroups places constraints on theories of what generates conspiracy theorizing. We argue that traditional “monological” believers are only the tip of an iceberg of commenters. Neither simple irrationality nor common preoccupations can account for the observed diversity. Instead, we suggest, those who endorse conspiracies seem to be primarily brought together by epistemological concerns, and that these central concerns link an otherwise heterogenous group of individuals.

## Introduction

Alexis de Tocqueville wrote that the American commitment to freedom of association prevented the formation of conspiracies (de Tocqueville, 1831). Considering recent political discourse, this seems optimistic. Indeed, conspiracy *theorizing* has long been a part of American politics (Hofstadter, 1964). The role of conspiracy theorizing has been intensified by a move to online discussion forums. The open discussion promoted by these forums forms a key mechanism for the spread of misinformation, including false conspiracy theories (Dunn et al., 2015; Zhou et al., 2015; Del Vicario et al., 2016). Conspiracy theories are not socially innocuous: aside from obvious political effects, endorsement of conspiracy theories is associated with rejection of science and unwillingness to donate to prosocial causes (van der Linden, 2015).

Numerous psychological accounts purport to explain the attraction of conspiracy theories. Conspiracy endorsers are said to be driven by feelings of powerlessness and lack of control (Whitson and Galinsky, 2008); to crave explanation in a fundamentally confusing world (Keeley, 1999); to be motivated by background political beliefs (Kahan, 2015); to seek social individuation (Raab et al., 2013); or to be misled by the “echo chambers” of online opinion (Bessi et al., 2015; Del Vicario et al., 2016).

One key line of research has characterized conspiracy believers as favoring a “monological” belief system (Goertzel, 1994; Swami et al., 2011; van der Linden, 2015), in which every event is connected to every other event. According to the monological account, individuals may begin with a particular instance of conspiratorial thinking. Then, through engagement with other ideas and through socialization with other conspiracy theorists, a particular mindset or worldview develops in which all historical and contemporary events can be explained in terms of hidden conspiracies.

As this is not a typical reaction to encountering conspiracy theories, monological theories typically attempt to ground the tendency toward a monological belief system in differences in personality traits, aberrant psychological processes, or other cognitive factors. So for example researchers ask “who tends to believe [conspiracy theories], and why” (Leman, 2007, 36), or inquire into the “psychological origin” of conspiracy theories (Swami et al., 2014, 573), or discuss the “similar underlying psychological processes” that are common to conspiracy believers (Van Prooijen et al., 2015, 2).

The monological view certainly captures something important about the popular conception of conspiracy theorists. Yet it remains unclear the degree to which it adequately characterizes the bulk of conspiracy endorsers. An alternative perspective maintains that conspiracy theorists are a psychologically heterogenous group. The appearance of a monological belief system is a twofold artifact. For one, only the most vocal and colorful conspiracy theorists are likely to come to public attention, obscuring a larger but more circumspect majority. For another, conspiracy theorists may appear in aggregate to believe all manner of things, just because an interest in conspiracy gives them common ground for interaction. Online conspiracy forums may be like online sports forums: aggregating over posters might give the appearance of fans who root for every team, even though most individuals favor only one. Similarly, setting aside the small group of monological theorists, it may be that no individual endorses a broad range of conspiracy theories.

Call this the *iceberg model*: it claims that monological believers are merely a small visible tip of a much larger heterogenous group. The distinction between the two models is important. On the monological model, a tendency to believe conspiracy theories is the primary explanatory construct for why conspiracy theorists exist (though this tendency itself may be explained in various ways). On the iceberg model, monological belief systems exist, but they primarily explain only why some conspiracy theorists come to be noticed. They say little about conspiracy theorizing itself.

The difference between the two positions matters in part because each has a different perspective on the rationality of conspiracy theorists. Most monological accounts assume that the process of conspiracy theorizing involves some fundamental rational flaw. Hence the need for a special construct in order to explain conspiratorial thinking. The details of this failing vary from account to account, but in general the picture is that conspiracy theorists fall short of normative standards for good evidence. So for example, conspiracy theorizing is claimed to correlate with rejection of science (Lewandowsky et al., 2013), or to be ameliorated by improvements in analytic thinking (Swami et al., 2014), or to have similarities with psychiatric conditions such as paranoid schizophrenia (Dagnall et al., 2015). Whatever the source, monological thinkers believe conspiracy theories merely because they cohere with other conspiracy theories, rather than on the basis of independent evidence (Goertzel, 1994). By contrast, the iceberg model predicts that failings of individual rationality are likely to be confined to the most visible members of a community (and perhaps not even all of those). Even if most conspiracy theorists happen to be wrong about the world, they are not distinctively irrational.

It is difficult to adjudicate between the two positions. Experimental studies can shed some light on the matter, but most use a convenience population of students and artificial tasks with questionable ecological validity. Examination of online comments to news stories (Wood and Douglas, 2015) gives more insight into the public arguments of committed conspiracy theorists, but selection bias remains a problem.

This study presents a novel way to investigate and quantify heterogeneity within a large online group of conspiracy endorsers. We used a publicly available collection of online comments from the r/conspiracy forum on the website reddit.com. Reddit is a collection of online forums (“subreddits”), most of which are user-run and moderated. Reddit is a popular site, with web traffic consistently ranking in the top 10 of US websites. While most forums are comparatively anodyne, Reddit's historical commitment to freedom of speech has allowed fringe communities to flourish. Reddit makes its comments available to researchers through a public API, and a comprehensive dataset covering nearly 8 years of comments is available for study.

We used this dataset to test three predictions of the iceberg model. First, we predicted that there would be substantial heterogeneity within posters to r/conspiracy. That is, we predicted that overall there would be numerous empirically derivable subgroups, each of which had distinct interests. This is in contrast to the prediction of the monological model, which portrays conspiracy endorsers as an essentially homogenous group. For example, Sunstein and Vermeule's (2009) theory of “conspiracy cascades” appears to predict that any conspiracy theory which gets traction at all will spread rapidly throughout a group, regardless of its content.

Second, we predicted that there should be posters within r/conspiracy who showed something like a monological belief system. The key feature of a monological belief system is that belief in one conspiracy predicts belief in a wide variety of others (Swami et al., 2011). The iceberg model predicts that the group of monological believers would be vocal but small: that is, they would be responsible for a disproportionate number of posts, but a relatively small number of the total posters.

Third and finally, the iceberg model predicts that there are a significant proportion of non-monological posters for whom there are no systematic linkages between how much they write and which conspiracies they discuss. The monological view, by contrast, predicts that the more conspiracy theorists talk about anything, the more they talk about everything. This can be tested by looking at correlations between how much people write and the sort of things they write about.

A final methodological point above is worth noting. A difficulty with using any large dataset of online discussions is finding a way to adequately quantify the contributions of individuals in an objective manner. In order to test the above predictions, we use a novel data-driven approach to building and analyzing the contributions of individual forum posters. We thus intend what follows to be not just a test of the iceberg model, but also a proof of concept of several techniques for deriving psychological conclusions from large online datasets. We thus follow in the footsteps of other authors (De Choudhury et al., 2016) who have used the reddit dataset to explore psychological motivations.

## Methods

### Ethics statement

Ethics approval was waived by the Macquarie University Research Office as we used only publicly available comments harvested via the Reddit API in accordance with the terms of service. All comments to the r/conspiracy forum were publicly available at the time of data collection (first quarter of May 2015). The “sample comments” extracted below were deliberately obfuscated to prevent their attribution to a particular user.

### Dataset and preprocessing

We used a publicly available dataset containing 1.7 billion comments and associated metadata spanning from October 2007 to May 2015. This includes nearly 2.25 million comments to r/conspiracy, made by about 130,000 distinct authors.

Reddit posts consist of an initial “link” post—typically a link to another website—followed by nested comments underneath. Our dataset contains only comments, which tend to be more substantial overall. For each comment, we removed those made by high-posting bots and pre-processed the text to remove common words, quoted text, and non-linguistic features (the Supplemental Appendix contains full details).

For the purposes of text analysis, a *document* is any collection of text to be analyzed as a unit. A *corpus* is an unordered collection of documents.

As we were interested in making inferences at the level of the author rather than the comment, we combined each author's comments into a single document per author. Our assumption is that by analyzing the sum contribution of an individual's comments to the conspiracy forum, we can make more accurate inferences about individual motivations and interests. One potential problem with this approach is that many authors make very few comments. Low-posting authors tend to give little information about their interests. To mitigate this, we removed authors who posted fewer than the median number (3) of comments.

The corpus was then processed to give a term frequency-inverse document frequency (tf-idf) representation of each author's combined contributions to the r/conspiracy forum. A tf-idf representation gives a normalized measure of the importance of particular words in characterizing a document. The tf-idf score for a term in a document is highest when that term appears infrequently across the corpus but frequently within the document, and thus is relatively distinctive. The tf-idf score is widely used in information retrieval, and can be seen as a method for determining the relevance of a particular term for the content of a document (Wu et al., 2008).

### Topic modeling

In order to give an objective, data-driven model to the reddit dataset, we built a *topic model* of r/conspiracy. Topic modeling is a method for representing each document in a corpus as being generated by a variety of distinct topics, each of which consists of a weighted set of words. Topic modeling techniques have become popular because they allow unsupervised dimensionality reduction: much of the variability in a large corpus like the r/conspiracy comments can be captured by a relatively small number of topics. The topics generated can be represented by a weighting on a set of words. Experimenters can easily interpret the model by an examination of the top words for each topic.

As topic modeling has its roots in automated information retrieval, most uses focus on the categorization of individual documents by sorting them into semantically similar clusters for later retrieval and analysis. However, one can equally well use topic modeling to explore the psychological motivations of the authors who make the comments. That is our approach: having fitted a topic model to each author's comments, we interpret it as an (abstract and idealized) model of the underlying psychological processes of the authors involved. Documents (in our case, each author's aggregated comments) are represented as a mix of different topics, allowing for fine-grained categorization and analysis.

To construct the topic model, we used non-negative matrix factorization (NMF), an algorithm developed to find common parts of pictures and which is well-suited to corpus analysis (Lee and Seung, 1999; Stevens et al., 2012). NMF takes a matrix with *a* different entries (one for each author) and *v* features (one for each term). It then factors it into a *t* × *v topic-word mapping* **H** and a *a* × *t document-topic mapping* **W**. The dot product of the two matrices approximates the original tf-idf matrix.

Intuitively, each entry in the topic-word mapping represents a weighted set of words that are a common “part” of many comments, and the document-topic mapping shows how each author combines those parts to make their combined contribution. The particular entry *W*_*at*_ is the *topic loading* for an author *a* on a topic *t*, and represents how important a particular topic is for reconstructing an author's combined comments.

NMF is similar to topic modeling by the more familiar Latent Dirichlet Allocation (LDA). Both decompose a bag-of-words representation of a corpus into a set of topics and a set of weightings by documents on those topics. LDA estimates a probabilistic generative model of the corpus, while NMF attempts to decompose a matrix representation directly. We chose NMF for two reasons. First, LDA has a number of hyperparameters which need to be tuned for a specific use. The topics generated are quite sensitive to these parameters. This makes LDA more flexible, but at the cost of making it more difficult to justify the choice of any particular setting. NMF, by contrast, has a widely-accepted default starting point (Boutsidis and Gallopoulos, 2008), leaving only the topic number *t* for the experimenter to chose. Second, a primary advantage of LDA over NMF—that the models can be updated as new information comes in—was irrelevant for the present case, as we possessed the entire corpus in advance.

The parameter *t* gives the number of topics, which must be chosen by the experimenter. The best choice of topic number depends on one's theoretical concerns (Von Luxburg et al., 2012). As our goal was to find common themes hidden among a group of superficially similar commenters, this argued for a comparatively small number of topics, compared to uses which seek to make fine-grained classifications of documents. Further, the larger the number of topics, the more likely one would fit on idiosyncratic features of individual authors; smaller topic numbers are thus a better test of heterogeneity.

Choosing too small a topic number tends to result in topics that overlap and run together different themes. To optimize between these two demands, we examined a quantifiable measure of the similarity of the derived topics to one another for a range of topic models. We constructed an NMF model for *t* = 12, 15, …90 and then calculated the mean cosine similarity between each pair of topics in the generated model. We chose *t* = 48 as it was the point where the mean cosine similarity began to flatten out (see Figure S1), suggesting a lack of substantial overlap between topics. The full 48-topic NMF model used for subsequent analyses is shown in Table S1.

Compared to other topic modeling algorithms, NMF has a reputation for creating topics with more diverse quality (Stevens et al., 2012)—the best topics are quite coherent and consistent, while other topics are more diverse. However, we think that the present study offers a more nuanced view. Many “rhetorical” topics are distinguished not by the pre-existing semantic similarity of the words to one another or their co-occurrence *per se*, but rather by the fact that they distinguish similar ways of talking *about* other topics. Topic 44 (for example) contains a set of words pertaining to reading articles both in print and online. These need not co-occur, and may even partially exclude one another, while still representing an intuitively cohesive way of talking about evidence.

### Subgroup clustering

We predicted that r/conspiracy would contain subgroups of authors whose posts differed in meaningful ways in their loadings on the extracted topics. To test this hypothesis, we looked at clusterings which categorized the authors in r/conspiracy into subgroups by using authors' complete topic loadings as the basis for a k-means clustering.

K-means is a widely-used clustering method. Conceptually, it partitions data by finding the best *k* different points (in this case, in the 48-dimensional topic space) and assigning each observation to the closest point in a way that minimizes the residual variance. Each of the *k* points is the mean of the observations assigned to it; thus each of the *k* points can stand as a representative of the cluster as a whole.

Establishing heterogeneity requires demonstrating two things. First, we show that there is latent structure within the group—that is, that clustering was a reasonable strategy. K-means requires specifying a target *k* and always returns a solution, so additional work is needed to show that the clustering solutions chosen are plausibly tracking real structure in the underlying data. Second, we needed to determine a plausible value for *k*. We did so by looking at values that seemed to do better at accounting for the data than nearby rivals. We note that the clustering solution presented is only one of several possible: large sets can often be clustered in distinct and equally good ways. Our selection procedure found clusters that were both real and theoretically interesting. As our goal was to establish substantial heterogeneity in posting patterns, we took this to be sufficient evidence to that end.

**Figure 1:**
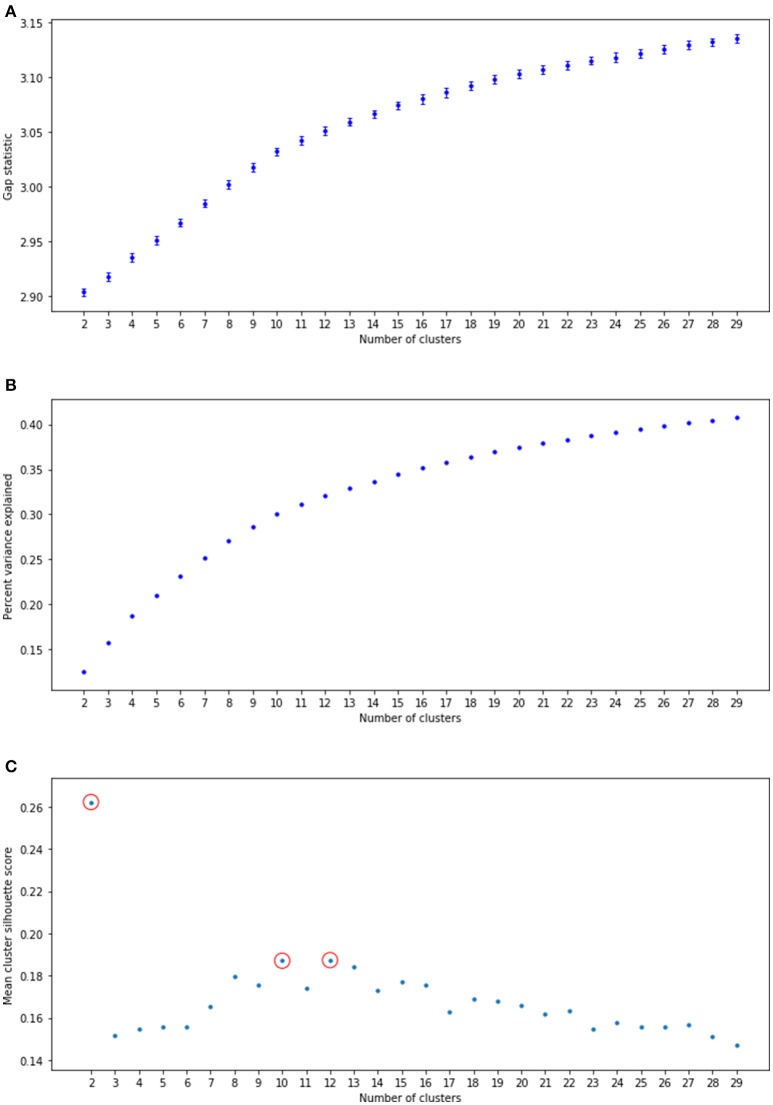
Statistics for a range of cluster solutions. **(A)** Gap statistic, following Tibshirani et al. (2001). Error bars indicate 6 standard deviations away from the null distribution. **(B)** Percent variance explained for a variety of clustering solutions. **(C)** Mean clusterwise silhouette score. Red circles indicate local maxima.

For the first step, we calculated the gap statistic for a range of clustering solutions *k* = 2…30, taking the average of 20 iterations for each. The gap statistic measures the difference between the within-cluster sum of squares for the actual clustering solution with the within-cluster sum of squares for a random dataset with points evenly distributed with the same bounds along each dimension (Tibshirani et al., 2001). This random dataset serves as a null model against which the improvement against real data with the same number of clusters can be tested. The gap was positive and greater than 6 standard deviations above 0 for each cluster solution (Figure 1A), suggesting real latent structure.

As the gap statistic did not itself suggest an optimal number of clusters, we next calculated the percentage of variance explained (PVE) for each solution. We observed an elbow in the range of 8–14 clusters (Figure 1B). Calculation of the silhouette score (Rousseeuw, 1987) suggested that there were local maxima at 2, 10, and 12 clusters (Figure 1C). The 2-cluster solution had a low PVE, so between 10 and 12 we chose the larger of the two. To further ensure that *k* = 12 was a reasonable cluster solution, repeated split-half Linear Discriminant Analysis (LDA) was used to verify cluster discriminability. The resulting mean classification accuracy of 89% (SD 0.002) is significantly different from expected chance performance of 0.36 (*p* < 0.001), suggesting that the derived subgroups do pick out distinguishable clusters in topic space.

### Subgroup statistics

For the entirety of r/conspiracy and each of the 12 subgroups, we calculated number of comments, number of authors, and mean number of comments by author. In addition to these basic descriptive statistics (Shown in Table 1), we examined two other measures to characterize the posting patterns of each subgroup relative to the whole.

**Table 1:** Basic Descriptive statistics for r/conspiracy and each subgroup. P/T = posts by group as percentage of total posts in r/conspiracy.

**Group**	**Posts**	**P/T**	**Authors**	**A/T**	**P/A**
r/Conspiracy	2253494	1.00	129829	1.000	17.36
Skeptics	21131	0.01	2003	0.02	10.55
Anti-Imperalists	111601	0.05	7625	0.06	14.64
Anti-Authoritarians	34700	0.02	2311	0.02	15.02
True Believers 1	531056	0.24	3650	0.03	145.49
Patriots	13986	0.01	880	0.01	15.89
Truthers	57907	0.03	1268	0.01	45.67
Psuedoscientists	163217	0.07	6523	0.05	25.02
True Believers 2	910116	0.40	2324	0.02	391.62
Anti-Semites	92532	0.04	1453	0.01	63.68
Indignant	47196	0.02	3011	0.02	15.67
Redditors	45091	0.02	3108	0.02	14.51
Uncategorized	124299	0.06	19146	0.15	6.49

*A/T, authors as percentage of total authors; P/A, posts per author*.

First, to visualize and characterize similarities and differences between subgroups, we looked at the topics on which members of a subgroup tended to load most highly. As our primary concern was looking for heterogeneity, we examined loadings on a subset of the most variable topics for each of the subgroups. We first selected the 15 subtopics which varied the most between subgroups by determining the standard deviation of the loadings by subgroup and then choosing those with the highest standard deviation. For each of the 15 selected topics, a significance level of *p* < 0.01 (uncorrected) was determined by a Monte Carlo method. The full document-topic mapping was shuffled and subgroups the size of the smallest subgroup were randomly selected; the mean loading on the topic was then calculated. This was repeated 1,000 times for each topic and used to create upper and lower bounds for significance.

Second, in order to characterize potential common interests both overall and within subgroups, we examined individual author's topic loadings as a function of how much they posted. A positive correlation between a topic loading and quantity of posting indicates that an author uses the words in the topic more *frequently* and *distinctively* the more they write. To quantify this, we examined the correlation between a user's loading on topics and the logarithm of each user's combined overall comment length [by character count, which correlates highly with both word count (*r* = 0.99) and number of comments made by the author (*r* = 0.91)]. The logarithm was used as comment length varies over several orders of magnitude. To account for a general correlation between topic loading and length across all topics, we generated 10,000 pseudo-topics by randomly selecting one of the 48 loadings for each author, and used this to calculate the mean and 95% uncorrected confidence interval for the correlation between loading and log length (*r* = 0.21 ± 0.005). Observed correlations above or below this interval were considered significantly different from the mean.

### Aggregate comments and tags

To facilitate discussion, we assigned each subgroup a short name based on their distinctive posting patterns and inspection of the most frequent posters' comments. In order to create a representative but anonymous sample comment, we chose comments from the top 2–4 posters in each subgroup, aggregating representative comments. We altered the grammar and word choice enough that a search would not reveal the original posters. Each poster has considerable variability in their own posts, but the quotes are representative of the whole.

Subgroup 11, the “uncategorized” group, contains a mix of low-posting contributors, missed bots, and other difficult-to-categorize posters. While there is undoubtedly structure in this group which might be extracted with further analysis, the noisy nature of the clustering means that we have omitted it from further discussion.

## Results

### Hypothesis 1

Hypothesis one was that there would be numerous empirically derivable subgroups, each of which had distinct interests. As shown in Figure 1, there was latent structure that facilitated a clustering solution.

**Figure 2:**
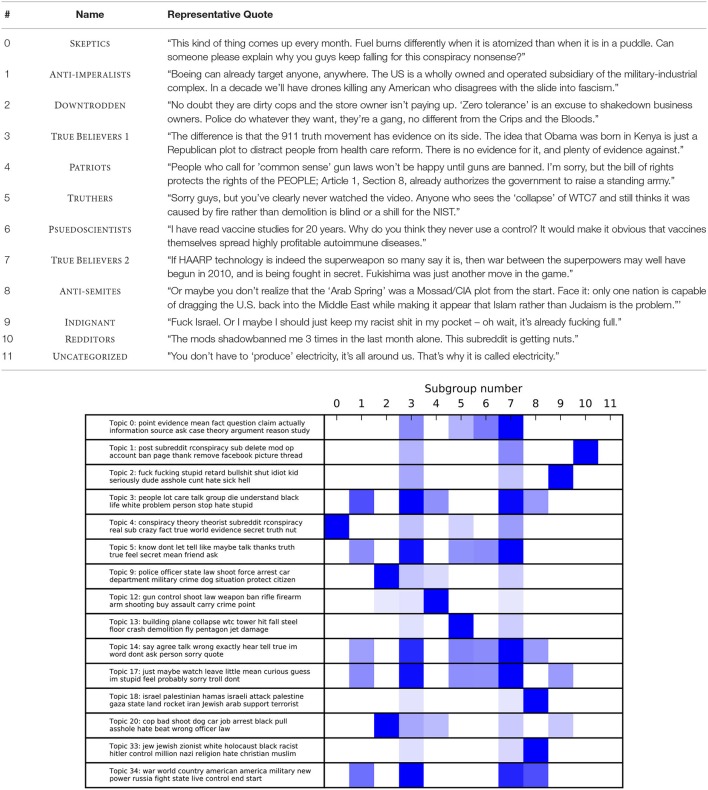
**(Top)** Subgroup, descriptive label, and aggregated quote (see text for details). **(Bottom)** Subgroup loadings on the most variable topics. Topics are represented by the top 15 words per topic. Loadings significantly above the mean are shown in blue (*p* < 0.01 uncorrected). Loadings are normalized per topic, with most saturated blue = highest loading for that topic.

This was borne out by the topic model. As shown in Figure 2 (and more fully in Table S1), the topic model gave intuitively interpretable topics. Some topics clearly pick out particular “topics” in the ordinary sense of the term: topic 33 contains words primarily relating to anti-semitism, while topic 34 contains words about American military power. In contrast, topics like 3, 5, 14, and 17 also appear to be primarily rhetorical in nature: that is, they capture something about how individuals discuss conspiracies, rather than specific content of conspiracies. Loadings on these topics vary across groups, albeit less dramatically. These topics seem to contain words that play an important role in mediating ingroup identification along with agreement and disagreement, suggesting that such mediation is differentially important for different groups. Finally, and notably, topic 0 concerns not the details of conspiracies but rather discussion around evidence and argument more generally.

The top of Figure 2 shows the mean loadings by subgroup on the 15 most variable topics. Mean topic loadings reveal substantial between-subgroup differences, as well as differences between individual subgroups and r/conspiracy as a whole. The bottom of Figure 2 shows a descriptive tag and aggregate comment for each group. The descriptive tags will be used in what follows, placed in small caps to emphasize that they are convenience labels.

In general, three patterns are seen. First, there are the two sets of True Believers, which load highly on most topics. Comment inspection suggests differences between these subgroups—very roughly, group 3 cares more about historical events while group 7 is willing to entertain more speculative conspiracies involving things like UFOs—but we will consider them together.

Second, there are groups who are distinguished by very high loadings on a single topic and average/low loadings on the others. Some of these are what we will call traditional “thematic” concerns: Anti-semites are concerned with Jews and Israel; Patriots by constitutional issues, and especially gun control; Truthers by conspiracy theories around the 9/11 attacks; Anti-Authoritarians by police use of force against civilians, and Anti-imperalists by the consequences of American power more generally.

Also of note in this pattern are the Pseudo-scientists, who are concerned with quasi-scientific topics such as anti-vaccination advocacy or chemtrails. This subgroup brings up an important point: topic modeling does not distinguish between those who endorse a conspiracy and those who argue with conspiracy theorists. Further, there is considerable debate even among True Believers about which conspiracy theories are worth endorsing. While this is an important caveat, it is a minor one. For one, comment inspection reveals that most subgroups are primarily composed of endorsers: those who are generally unimpressed by conspiracy theories are lumped by the algorithm into the Skeptics. For another, the fact that there are linguistically separable patterns of argument that cluster by topic is interesting.

The third and final pattern was less expected. There are groups who are distinguished by more “rhetorical” topics. The most notable among these are the Indignant, which are distinguished by high loading on a topic consisting of angry invective. What distinguishes the Indignant, in other words, is not what they talk about but how they talk about it. Similarly, Redditors are marked by discussion of Reddit itself and associated social drama, rather than particular themes.

### Hypothesis 2

Hypothesis two was that there would be a subset of posters who fit the monological pattern, and that these would form a relatively small but vocal minority. This is borne out by the data. As shown in Figure 2, the two groups of True Believers load significantly on most of the chosen topics. They thus appear to come closest to the classic picture of a monological belief system.

The True Believers are responsible for a disproportionate number of comments. Although only 5% of the posters in the subreddit, they made 64% of the comments in the forum. These posters thus have substantially higher posts per author than other subgroups. The average number of posts per author in r/conspiracy is 17, while the two subgroups averaged 145 and 392 posts per author. Further, some individuals in the group also write a staggering amount. The highest-posting author in True Believers wrote 896,337 words (post-processing) across over 18,000 posts—roughly twice the combined length of the *Lord of the Rings* trilogy.

### Hypothesis 3

Third and finally, the iceberg model predicts that (monological posters aside), there should be no systematic correlations between how much people write and which conspiracies they discuss.

**Figure 3:**
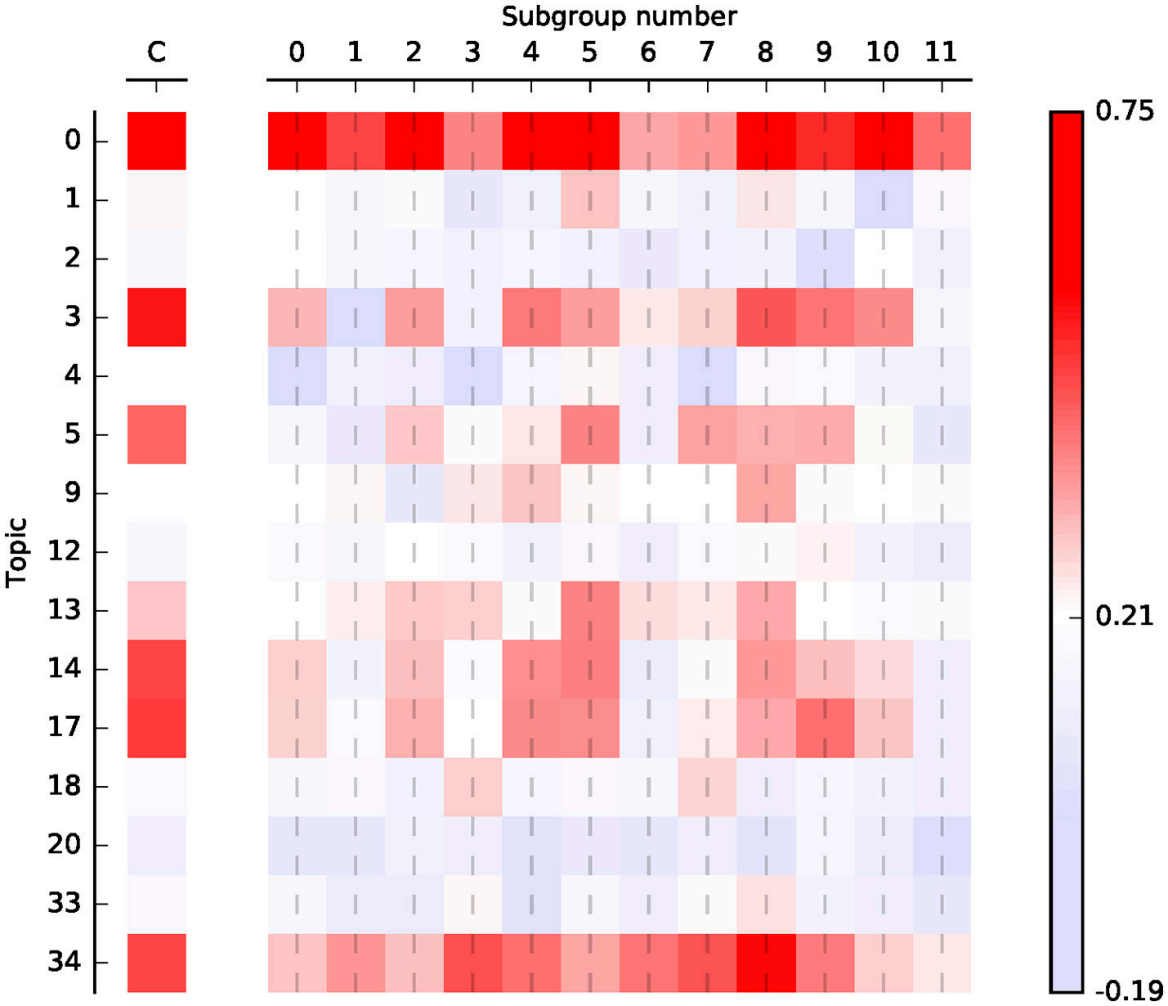
Correlations between log comment length and loading for selected topics, corrected for overall correlation between mean loading and length. Correlations shown for conspiracy overall (“C”) and for each of the 12 subgroups. Correlations significant to *p* < 0.05 uncorrected around the average random correlation of 0.21.

Figure 3 shows the correlation between topic loadings and log comment length. (Raw numbers can be found in Table S2). This shows which topics become more important the more people post. Consider, for example, the Anti-semites, who load heavily on the two topics having to do with Judaism. The correlation with log length and these two topics is only slightly above average, as you might expect: group membership is defined in part by placing a high importance on these topics no matter how often one posts. On the other hand, Anti-semites show a high loading with (e.g.,) topic 13, suggesting that 9/11 conspiracy theories become more important for this group the more they write. Conversely, the opposite pattern is also shown—most subgroups are slightly *less* likely than average to talk about gun control the more they write.

Along the rows, two striking commonalities emerge. Topic 0, which concerns evidence, belief, and argument, has the strongest correlations of the group, ranging as high as 0.75 in the subgroups and 0.73 overall. Topic 34, concerning American military power and foreign policy, has a similarly high pattern of correlation (as does topic 35, not pictured here).

## Discussion

The iceberg model made three predictions: that posters in r/conspiracy have distinguishable sets of interests, that individuals with a monological set of beliefs are a small but vocal minority within the population, and that no particular set of conspiracy theories would correlate highly with increased posting. The first two predictions were verified by the dataset. The third received mixed support. Of the two topics which appear to significantly correlate with post length, only one (topic 34) correlates highly with comment length in all subgroups. This topic contains words concerning US foreign policy, which makes it difficult to determine the order of explanation. The US is a powerful country; it may simply be a convenient touchstone for a variety of otherwise different interests.

A core assumption of our approach was that topic modeling of aggregate user comments was a reasonable proxy for psychological states of the authors so modeled. On the one hand, this is a difficult background assumption to independently validate. Previous uses of the reddit dataset to extract psychological states have focused on subsequent posting behavior (De Choudhury et al., 2016). We have no similar link. On the other hand, we assume that it is relatively uncontroversial to take what people say to be reasonable proxies for what they are thinking about. Reddit posting is uncompelled; the fact that someone choses to write a fair bit about (say) Israel's purported involvement in 9/11 suggests that this is a topic that is close to their heart.

Further, all things being equal, people who use what appears to be extremely angry or conciliatory language are probably angry or conciliatory, respectively. In the process of drawing up the labels for Figure 2, we manually inspected a large number of comments by members assigned to particular subgroups, and confirmed that the topics upon which they loaded most highly did reflect the topics that they appeared to be concerned with. Finally, the fact that authors do not appear to self-segregate by thread within the forum suggests that our measures are tracking primarily facts about authors and their individual interests, rather than about the structure of the forum discussion itself.

A further methodological point is worth re-emphasizing. Our topic model fit a tf-dif representation of the corpus. Hence extracted topics capture not merely which terms tend to co-occur (which may be a function of what is discussed), but which terms tend to be distinctive of individual authors' contributions. This is additional reason to believe that topic loadings track what is distinctive about an author's contribution to the forum.

Hence we take it that our method adequately tracked something about individual authors' interests and beliefs. The support for the iceberg theory suggests that the monological theory of conspiracy theorizing is at best incomplete. With that in mind, we turn to the broader theoretical question of what might drive conspiracy theorizing.

### Language use: four interpretations

Our study showed both commonalities and differences between subgroups posting in r/conspiracy. What, if anything, can one say about conspiracy believers as a whole? We evaluate four classes of psychological theory about conspiracy endorsement in light of these findings.

#### Irrationality

It is common to suppose that conspiracy endorsement is a sign of *prima facie* irrationality. The idea of a “monological belief system” is typically introduced as the idea that belief in one conspiracy predicts belief in different, apparently unrelated theories (Swami et al., 2011; Wood et al., 2012). Many have noted the power of conspiracy theories to give broad, unifying explanations at the expense of plausibility (Keeley, 1999). This suggests a particular picture: the conspiracy theorist is one who believes that *everything* is connected. As this is antecedently implausible, irrationality seems like the best explanation.

Yet we think that a move to attribute pathological irrationality would be hasty. First, as we noted above, it is easy to overgeneralize from committed posters. But second, and importantly, the strong correlation between length and topic 0 suggests that posters are concerned above all with evidence and argument. Contrary to the unflattering picture presented by many authors, on which conspiracy endorsers are merely gullible or deluded, we note that conspiracy theorists in fact spend an inordinate amount of time discussing *why* they believe what they believe (Hofstadter, 1964).

Here we enter into delicate territory. There is a common assumption that the content of conspiracy theories is—perhaps by definition—irrational to believe. Conspiracy theories about reptilian overlords or the Queen of England can seem downright delusional. Yet it cannot be irrational *tout court* to believe that the CIA meddles in international affairs, or that scientists are experimenting on vulnerable populations without their consent, or that the US government secretly monitors the activities of dissident groups. These things have all happened. Thus we think it unlikely that belief in conspiracy theories is straightforward evidence of irrationality. Indeed, as Dagnall et al. (2015) note, there are situations in which skepticism about official narratives is arguably more adaptive than simple acceptance; this explains the numerous important differences between clinical populations and conspiracy believers.

Finally, as one of our sample quotes for the True Believers suggests, even voluminous posters often see themselves as discerning, rejecting as many conspiracies as they accept. Tanya Luhrmann noted a similar phenomenon among practitioners of modern witchcraft, suggesting that among marginalized communities, internal debate is often as important as agreement (Luhrmann, 1991).

#### First-order content

Another possibility is that conspiracy believers are distinguished by particular sets of common beliefs. This is arguably the everyday picture—a conspiracy theorist just is someone who believes in conspiracy theories, and there is little more to say.

The evidence for heterogeneity shows that there are not necessarily any common first-order beliefs shared by all conspiracy theorists. Noteworthy is the ability of our method to distinguish between groups like Anti-Authoritarians and Patriots. Both are concerned, in some broad sense, with government violence and abuse of power. But they show different patterns of topic loading and different correlation patterns, suggesting important distinctions between groups who may well discuss the same events for superficially similar reasons.

What may be the case, and is consistent with our data, is that individuals have particular interests to which they connect events if they can. This may represent a different sense of “monological” belief, often confused with the more overarching sort in which everything connects to everything. Goertzel himself thought that the characteristic of conspiracy theorizing was something more on the order of an *idée fixe* rather than a grand narrative. As he put it:

Monological conspiracy thinkers …offer the same hackneyed explanation for every problem—it's the conspiracy of the Jews, the capitalists, the patriarchy, the communists, the medical establishment, or whatever. In these cases, the proof which is offered is not evidence about the specific incident or issue, but the general pattern; for example, the *X* conspiracy has been responsible for all of our other problems, so it is obvious that *X* must be responsible for this one as well (Goertzel, 1994, 741)

Rather than a dense holistic web in which everything supports everything else, there is a single focus to which other problems can be connected. Sometimes the two are difficult to distinguish: a belief that the Jews secretly run the world is easy to connect to many different problems. But some obsessions are less fertile. The idea that the US government is secretly conspiring to confiscate firearms, for example, might connect to a wide variety of current events but not to vaccination, or the moon landing, or the death of Princess Diana.

It is worth noting that many “academic” conspiracy theories are of this sort. Consider Gavin Menzies' (2003) claim that Chinese treasure fleets landed in Nova Scotia during the Ming Dynasty. This has been widely debunked, and Menzies' narrative style has been explicitly compared to that of conspiracy theorists (Goodman, 2006). Yet while Menzies plays fast and loose with evidence that might conceivably support his hypothesis, and is keen to accuse mainstream scholarship of coverups, he has little interest in events that *don't* have bearing on the Ming treasure fleets.

#### Second-order content

Another (not exclusive) possibility is that conspiracy theorizing is driven largely by what we will call *second-order* concerns: that is, concerns about how one arrives at first-order conclusions. Wood et al. (2012, 1) suggest that learning about one conspiracy may encourage belief in others because “Even though the perpetrators may be different in each case, the fact that one massive, sinister conspiracy could be successfully executed in near-perfect secrecy suggests that many such plots are possible.” The conspiracy theories so endorsed need not bear any particular relationship to one another. Indeed, it is possible that they might be mutually inconsistent (Wood et al., 2012). Wood et al. note a positive correlation between endorsing the statement that Osama Bin Laden was dead before US soldiers arrived and endorsing the statement that he is still alive. One might have thought those to be difficult beliefs to hold simultaneously. However, this correlation is mediated entirely by a more general belief that officials are engaged in some sort of cover-up.

The idea that some sources of information are unreliable cannot be an intrinsically irrational belief. As recent discussions of “fake news” emphasize, everyone believes that at least some media sources are systematically distorted. Epistemic responsibility thus involves keeping track of which are reliable. Indeed, we find substantial evidence that posters in r/conspiracy are concerned with general problems about epistemic responsibility. Recall that topic 0—dealing with evidence and argument–correlates strongly with log comment length for all subgroups. This suggests that the more authors talk, the more they care about evidence, belief and argument.

#### Rhetorical features

The above mostly concerns what conspiracy endorsers believe. Yet as Hofstadter's classic work put it, “Style has more to do with the way in which ideas are believed and advocated than with the truth or falsity of their content. It is the use of paranoid modes of expression by more or less normal people that makes the phenomenon significant…nothing really prevents a sound program or demand from being advocated in the paranoid style” (Hofstadter, 1964, 77). A fourth possibility, therefore, is that conspiracy endorsement is a function of *style* as much as substance.

The most obvious example of this are the Indignant, who tend to write short, profanity-heavy posts. Indeed, the Indignant raise an interesting possibility: some posters might *endorse* a conspiracy theory without fully believing it, as a way to express their background commitments. Someone might endorse the statement “The US was behind 9/11,” not because they have positive beliefs about what happened, but as a way to vent their anger about US domestic policy. Racist conspiracy theories may play a similar role. Similarly, the Redditors seem to be as much concerned with interpersonal drama on the site as they are with any particular conspiracy theory.

Finally, rhetorical topics may play a moderating role. Our methods are relatively coarse-grained, and within groups such as Truthers, there can be considerable disagreement (Wood and Douglas, 2015). Among our Truthers, topics such as 4, 5, 14, and 17 correlate well with topic length *and* load higher than average. These topics contain words that involve (among other things) moderating inter-group disagreement, providing evidence that such groups are diverse even internally. This suggests that conspiracy theories may be interesting to some in part because they can sustain that sort of interpersonal conflict, which in turn aids with personal individuation (Raab et al., 2013).

### Limitations and future directions

Several features and limitations of the present study are worth noting.

Topic analysis does not distinguish between people who endorse a conspiracy (in any sense) and those who argue with conspiracy theorists. Automated methods such as sentiment analysis for teasing out agreement and disagreement would be a useful addition to the present method. Similarly, there is no way to tell whether some posters have a more monological belief system than would be indicated by their postings but chose to be circumspect in what they discuss online. We suspect this is unlikely: r/conspiracy is as welcoming a forum as one might find for expressing conspiracy beliefs. However, individual differences in willingness to discuss topics may be a confounding issue, and one that deserves further study.

More broadly, we formed subgroups solely by looking at language usage. Other groupings are possible with the reddit dataset: one might look at network structure both within and outside of r/conspiracy, for example, by looking at relationships of mutual commenting. Examination of the network structure of other forums (Dunn et al., 2015; Zhou et al., 2015; Del Vicario et al., 2016) has given important insight about the dynamics of problematic beliefs as they spread through social networks, and would be a useful complement to the current investigation. Similarly, one might look at other places in which authors post, as an index of their overall interests outside r/conspiracy. That said, a strength of the current study is that it is able to distinguish structure without relying on network considerations. There is no evidence that subgroups self-segregate; indeed, threads tend to contain representatives from many different subgroups (Table S2).

## Conclusions

In contrast to the monological account of conspiracy theories, we have demonstrated the degree to which conspiracy endorsers differ. Psychologists have suggested a wide variety of different motivations for belief in conspiracy theories, including the need for explanations, the desire for control in a complex world, political extremism, the desire for simple explanations, and so on. Some of these do not appear to be especially problematic motives *per se*. Indeed some form of low-grade conspiracy theorizing is widespread (consider the kvetching that academics do about the nefarious plans of administrators and granting bodies).

Monological theorists are a small, but vocal minority within this online community. Were we to take r/conspiracy as a whole as a single author, that author would undoubtedly seem monological and paranoid. Yet very few authors within conspiracy fit a clearly monological pattern. We suggest that r/conspiracy looks monological in part because there are many different authors with different sets of concerns, each interacting with one another. Consider a thread about (e.g.,) secret CIA prison camps. One person might care about its relationship to 9/11, another might use it to fuel their anti-semitism, a third to make a point about gun control. Each gets what they need, and each contributes to the larger whole.

Ultimately, we doubt that there needs to be any particular set of psychological motivations which characterize conspiracy theorists. Some are irrational. Some are irate. Some are epistemically unlucky. Some are racist. Some are skeptical. We should not say that conspiracy theorists have overarching belief systems that encompass and unify a wide variety of different narratives. Instead, it may be the other way around: it is conspiracy *narratives* that are all-encompassing, pulling in a diverse group of people who may have little in common with one another, each of whom can find what they need in a fragment of the larger tale.

## Author contributions

CK, PC, and VP designed the study. CK ran the analyses. CK, PC, and VP jointly interpreted the results, drafted the manuscript and did critical revisions.

### Conflict of interest statement

The authors declare that the research was conducted in the absence of any commercial or financial relationships that could be construed as a potential conflict of interest.
