# Supplementary material for: Topic Modeling Reveals Distinct Interests within an Online Conspiracy Forum
Source: Front Psychol. 2018 Feb 21;9:189. doi: 10.3389/fpsyg.2018.00189 (PMC5826393; doi:10.3389/fpsyg.2018.00189)
Supplement: Supplementary file 1 [file Presentation1.PDF]

Supplementary Material: Topic Modeling Reveals Distinct Posting Patterns Within an Online Conspiracy Forum

DATA PROCESSING

Dataset

We used a dataset made publicly available by the user 'Stuck_In_The_Matrix', who used the official reddit API to scrape a set of roughly 1.7 billion comments and associated metadata spanning from October 2007 to May 2015. (For more details see `https://www.reddit.com/r/datasets/comments/3bxlg7/i_have_every_publicly_available_reddit_comment/`). The set has nearly every public reddit comment made in the time period. This includes nearly 2.25 million comments to `r/conspiracy`, made by nearly 130,000 distinct authors.

Comments outnumber link posts by roughly 12:1, and link posts rarely contain more than a URL. We hand-checked the top 10 most commented-upon links in our dataset, and only one contained substantial original text. Hence only comments are included in our dataset and subsequent analysis.

Preprocessing

Reddit allows posts by small automated programs known as 'bots', which can skew descriptive statistics. To remove account names associated with bots, we first looked at each poster in a target set of subreddits (including `r/conspiracy`) and calculated the number of other subreddits in which they posted (their *forum diversity*). A list was compiled of usernames whose forum diversity was more than 15 standard deviations above the mean. Manual inspection revealed that every member of this list was probably a bot, whereas more aggressive cuts also included posters who were clearly human. This was combined with a list of usernames corresponding to known bots posted on reddit itself. (From `https://www.reddit.com/r/botwatch/comments/1xojwh/list_of_320_reddit_bots/`)

The 466 authors in the set were excluded from subsequent analyses. This procedure erred on the side of including bots rather than eliminating human posters. While the remaining set contains some bots, the combination of forum diversity and known bots eliminates the

vast majority of automated posting, and the remaining bots should make for only a minor skew on the descriptive statistics. Of note, the remaining bots (such as there are) seem to be concentrated in the 'noisy' subgroup 11, about which we make no specific claims.

After extracting the comments in `r/conspiracy` from the larger dataset, the comment text itself was preprocessed in several steps. Comments with authors or text bodies which had been subsequently deleted were removed. Whole lines which were preceded by markdown quotation indicators were removed, as were URLs and formatting escape codes. Words were then tokenized and converted to lowercase, and punctuation and non-alphabetic characters were removed.

Words were tagged with part-of-speech using the python Natural Language Toolkit package (`nltk`, Bird et al. (2009)). Functional words such as pronouns and determiners were removed, leaving only nouns, adjectives, verbs, adverbs, and cardinal numbers. What remained was then lemmatized with `nltk` using the wordnet lemmatizer Miller (1995). Any comment fewer than 3 words after preprocessing was eliminated from further analysis. Note that, unlike many studies, we did not eliminate short words, as there were semantically meaningful worlds of two and three characters that clearly belong in the final topics.

Because we are interested in drawing inferences about authors rather than individual comments, each author's comments were combined into a single document. This also required removing comments where the author was listed as '[deleted]', which occurs when a user closes their account but when preserving the comment tree structure requires keeping a placeholder for a comment they made.

The resulting corpus was transformed into a term frequency-inverse document frequency (tf-idf) representation. (This and subsequent steps used `scikit-learn`, Pedregosa et al. (2011)). A tf-idf representation represents individual documents as a collection of normalized word frequencies, each weighted by the overall frequency of the word in the corpus. The weighting means that words which are extremely common are given relatively little weight, while words which distinguish a particular document are weighted more. At this step any remaining common english stopwords and words that appeared fewer than 20 times in the corpus were also eliminated, leaving a final vocabulary of 30,327 words.

Data Sharing

Processed data for `r/conspiracy` is available as a 165MB archive at:

`https://cloudstor.aarnet.edu.au/plus/index.php/s/GRjLJxoxN99RIJe`

To ensure anonymity, the processed data contains neither author names nor identifiable comments.

SUPPLEMENTARY FIGURES AND TABLES

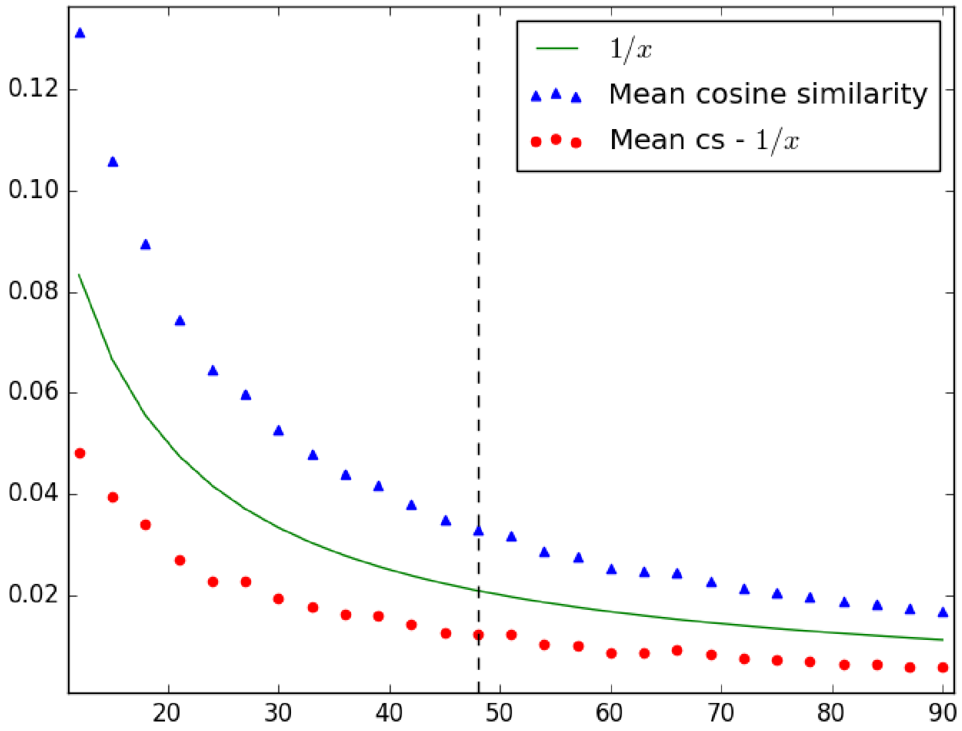

Figure S1: Mean pairwise cosine similarity for each vector in the topic-word mapping for a range of NMF topic numbers $12, 15, \ldots 90$. $1/x$ represents the theoretical lower bound of this value. Red circles indicate difference from the theoretical lower bound. Chosen topic number of 48 indicated by dashed line.

0	point evidence mean fact question claim actually information source ask case theory argument reason study
1	post subreddit rconspiracy sub delete mod op account ban remove page facebook thank picture thread
2	fuck fucking stupid retard bullshit shut idiot kid seriously dude asshole cunt hate sick hell
3	people lot care talk group die understand black life white problem person stop hate stupid
4	conspiracy theory theorist subreddit rconspiracy real sub crazy fact true world evidence secret truth nut
5	know dont let tell like maybe talk thanks truth true feel secret mean friend ask
6	video watch youtube fake camera saw footage game film minute explain view release documentary second
7	good bad job idea point luck great thank thanks damn feel movie evil sir stuff
8	guy picture talk hey shoot bomb crazy backpack run black actually lol stuff idiot white
9	police officer state law shoot force arrest car department military crime dog situation protect citizen
10	look picture photo fake image eye pic backpack maybe edit lot face left white bomb
11	government control citizen american federal power law state medium public terrorist corporation secret protect corrupt
12	gun control shoot law weapon ban rifle firearm arm shooting buy assault carry crime point
13	building plane collapse wtc tower hit fall steel floor crash demolition fly pentagon jet damage
14	say agree talk wrong exactly hear tell true im word dont ask person sorry quote
15	time long year day waste start hear watch spend hour tell ago live old new
16	really wow care stuff hope lot interesting big kind feel bad like watch thanks understand
17	just maybe watch leave little mean curious guess im stupid feel probably sorry troll dont
18	israel palestinian hamas israeli attack palestine gaza state land rocket iran Jewish arab support terrorist
19	read book interesting thank title write great actually thanks story stop stuff sound watch remember
20	cop bad shoot dog car job arrest black pull asshole hate beat wrong officer law
21	right wrong law freedom realize left amendment sound wing leave constitution speech citizen mean free
22	believe tell god truth hard lie story actually alien true religion theory crazy fake idiot
23	need help start stop change new feel page power upvotes dont let attention problem revolution
24	link thanks source click page site google thread provide share thank website info original check
25	make sense feel sick point fun difference statement sound bad decision wonder mistake person sad
26	kill child kid year die shoot innocent death terrorist dog dead murder civilian school family
27	think mean maybe agree exactly joke dont crazy idea probably word honestly big lot thought
28	happen year day actually event story holocaust ago let probably wait week attack remember exactly
29	want live hear exactly dont truth talk ask help change let free watch stop question
30	comment thread delete sub account mod ban edit shill subreddit rconspiracy reply op page user
31	come day mind hear truth hope year true let wait new start tell alien home
32	sure pretty im lot actually probably sound joke big funny talk stuff lol dude hear
33	jew jewish zionist white holocaust black racist hitler control million nazi religion hate christian muslim
34	war world country american america military new power russia fight state live control end start
35	use word water phone weapon google drug force internet site data term technology chemical company
36	vote party obama paul election president candidate ron republican voting change count voter win democrat
37	reddit mod sub ban user account page site admins subreddit new subreddits remove rconspiracy censor
38	thing bad lot kind sort change different hear exact funny probably life stuff important learn
39	yeah oh lol sound mean probably hear totally hell stuff haha dude definitely wait idiot
40	money pay tax bank year company buy dollar debt gold business loan free market spend
41	way feel life change best easy long start let help power agree live possible great
42	man love god oh thank life black great thanks lol live hear white yes real
43	news source story fox medium report watch hear cnn old tv site day page mainstream
44	article year old ago write title source mention author website wikipedia quote original google publish
45	work job day hard year live school company hour great tell kid life week home
46	try nice stop help hard tell start shill let troll understand maybe sound hide figure
47	shit holy fucking piece real stupid bullshit crazy lol oh dont dude dumb im sub

Table S1. 48-topic NMF model for `conspiracy`. Showing top 15 words for each topic.

**Group**	MLD
`r/Conspiracy`	7.2
Skeptics	7.3
Anti-imperalists	7.4
Anti-authoritarians	8.4
True Believers 1	6.4
Patriots	8.2
Truthers	7.7
Psuedoscientists	6.9
True Believers 2	5.9
Anti-Semites	7.3
Indignant	7.7
Redditors	7.2
Uncategorized	7.1

Table S2. *Mean link diversity* (MLD) for each subgroup. MLD measures how many other subgroups, on average, post to threads in `r/conspiracy` that authors in a subgroup post to. In our study, MLD can range from 1 (authors in the subgroup post only with authors in their own subgroup) to 12 (authors post in threads with representatives from every other subgroup.) A low MLD would suggest that members of the subgroup self-segregate within the forum, while a high MLD suggests the opposite.

	C	0	1	2	3	4	5	6	7	8	9	10	11
0	0.73	0.56	0.47	0.62	0.38	0.61	0.75	0.34	0.35	0.64	0.51	0.6	0.41
1	0.23	0.22	0.15	0.2	0.08	0.13	0.3	0.15	0.13	0.25	0.16	-0.01	0.17
2	0.16	0.22	0.17	0.15	0.14	0.15	0.14	0.09	0.14	0.14	0.03	0.22	0.13
3	0.54	0.32	-0.19	0.35	0.14	0.4	0.35	0.25	0.28	0.44	0.4	0.37	0.15
4	0.22	-0.0	0.13	0.12	-0.03	0.15	0.23	0.12	-0.08	0.17	0.18	0.14	0.12
5	0.43	0.16	0.09	0.29	0.2	0.25	0.38	0.11	0.35	0.33	0.33	0.23	0.08
9	0.21	0.21	0.23	0.08	0.25	0.3	0.23	0.21	0.21	0.34	0.2	0.21	0.19
12	0.16	0.18	0.16	0.22	0.18	0.15	0.18	0.11	0.18	0.2	0.23	0.15	0.11
13	0.29	0.22	0.24	0.29	0.28	0.2	0.39	0.26	0.25	0.34	0.21	0.19	0.19
14	0.47	0.28	0.15	0.3	0.18	0.37	0.39	0.11	0.2	0.35	0.31	0.27	0.11
17	0.48	0.28	0.18	0.32	0.22	0.37	0.37	0.13	0.24	0.33	0.41	0.3	0.12
18	0.18	0.17	0.17	0.14	0.29	0.15	0.17	0.17	0.28	0.12	0.15	0.14	0.11
20	0.11	0.07	0.07	0.13	0.11	0.04	0.09	0.08	0.11	0.06	0.15	0.1	0.03
33	0.18	0.16	0.1	0.1	0.23	0.04	0.16	0.11	0.2	0.26	0.15	0.11	0.08
34	0.47	0.3	0.36	0.3	0.46	0.41	0.34	0.41	0.45	0.56	0.4	0.28	0.25

Table S3. Numeric correlations for selected topics and log length; corresponds to figure 2 in main text.

REFERENCES

Bird, S., Klein, E., and Loper, E. (2009). *Natural language processing with Python* (O'Reilly Media, Inc.)

Miller, G. A. (1995). Wordnet: A lexical database for english. *Communications of the ACM* 38, 39–41

Pedregosa, F., Varoquaux, G., Gramfort, A., Michel, V., Thirion, B., Grisel, O., et al. (2011). Scikit-learn: Machine learning in Python. *Journal of Machine Learning Research* 12, 2825–2830
